# *Ponticolaalasanicus* sp. n. (Gobiiformes, Gobiidae) from the Alazani River Basin, Georgia

**DOI:** 10.3897/BDJ.11.e101095

**Published:** 2023-05-30

**Authors:** Giorgi Epitashvili, Bella Japoshvili, Levan Mumladze

**Affiliations:** 1 Ilia State University, Tbilisi, Georgia Ilia State University Tbilisi Georgia

**Keywords:** *Ponticolaalasanicus* sp. n., freshwater gobies, DNA barcoding, taxonomy, Western Caspian Sea Basin

## Abstract

**Background:**

The South Caucasus Region and Georgia, in particular, is a biodiversity hotspot and characterised by high diversity of landscapes and ecosystems, as well as high levels of endemism. At the same time, diversity of freshwater organisms in the region remains poorly studied, including fishes. The freshwater fish fauna of the South Caucasus Region consists of 119 fish species, of which 13 species belong to the order Gobiiformes. It should be noted that gobies are amongst the poorly studied taxa in Georgia and probably unknown/undescribed species still living in the Georgian freshwater ecosystems which requires further research.

**New information:**

*Ponticolaalasanicus*, a new species is described from the Alazani River, western Caspian Sea Basin, Georgia. It is distinguished from its congeners in the Caspian and Black Sea Basins by having the following features: dorsal fin with VI-VII spines and 15½-16½ branched rays, anal fin with 10½-12½ branched rays; lateral line with 48-55 scales; laterally compressed body with dark brown and black blotches - scales ctenoid; first and second dorsal fins almost touching with dorsal fins bases; head large, depressed, wider than deep, its length approaches almost 3.4th of standard length; nape scaled completely; cycloid scales cover upper part of opercle, cheeks noticeably swollen; snout longer than eye, eye diameter 4.5 times its head length; lower jaw slightly protruding; upper lip is uniform; pelvic disc short, elongated and flat, not reaching the anus; the pectoral fins extends vertically through first branched dorsal fin; caudal fin rounded. *Ponticolaalasanicus* sp. n. belongs to *P.syrman* group and it is separated by a minimum Kimura 2-parameter distance of 3.5, 3.6 and 4.8% from *P.syrman*, *P.iranicus* and *P.patimari*, respectively.

## Introduction

The family Gobiidae Cuvier, 1816 is one of the most diverse group of vertebrates and includes 257 valid genera with more than 2000 species (Fricke et al. 2022). Gobies are small-sized fish that mostly inhabit saline and brackish waters; few of them are found in freshwater habitats around the world (Kottelat and Freyhof 2007, Tutman et al. 2020). In Europe, freshwater gobies are most diverse in the Mediterranean, Black and Caspian Sea Basins (Kottelat and Freyhof 2007). The Black and Caspian Seas belong to the Ponto-Caspian biogeographical region (Vasil'eva et al. 2015), part of which is recognised as a Caucasus biodiversity hotspot (Zazanashvili et al. 2020). The freshwater fish fauna of the South Caucasus Region consists of 119 fish species, of which 13 species belong to the order Gobiiformes, which are united into two families and seven genera (Kuljanishvili et al. 2020). The genus *Ponticola* Iljin, 1927 is restricted to the Black and Caspian Sea Basins and does not enter the Marmara or Mediterranean Seas (Freyhof 2012), though the species *P.kessleri* has reached the northern seas and river systems via artificial channelling (Thiel et al. 2017). Within the South Caucasus and particularly in Georgian freshwaters, the genus *Ponticola* is most numerous and represented by four valid species: *P.constructor* (Nordmann, 1840), *P.cyrius* (Kessler, 1874), *P.gorlap* (Iljin, 1949) and *P.syrman* (Nordmann, 1840) (Kuljanishvili et al. 2020). Within this genus, six more species have been described in the last decades from the Caucasus and Irano-Anatolian biodiversity hotspots, including *P.rhodioni* (Vasil'eva & Vasil'ev 1994a) from the rivers flowing into the Black Sea, north of the Bzyb' Mountain Range and in the Kuban Basin, *P.rizensis* (Kovačić and Engín 2008) and *P.turani* (Kovačić and Engín 2008) from the south-eastern Black Sea Basin; *P.iranicus* Vasil'eva, Mousavi-Sabet & Vasil'ev, 2015; *P.patimari* Eagderi, Nikmehr & Poorbagher, 2020 and *P.hircaniaensis* Zarei, Esmaeili, Kovačić, Schliewen & Abbasi, 2022 from the southern Caspian Sea Basin (*Vasil′eva and Vasil′ev 1994b*, Kovačić and Engín 2008, Vasil'eva et al. 2015, Eagderi et al. 2020, Zarei et al. 2022).

During a recent field collection event in 2020 in eastern Georgia, we collected goby specimens from the Alazani River, which were preliminarily identified as *P.cyrius*. However, later CO1 DNA barcoding showed substantial differences with other known *Ponticola* species from Georgia and surrounding regions. Based on further detailed investigation of the collected material, in the following, we decide to provide a description of a new *Ponticola* species from eastern Georgia.

**Study area**

The Alazani River is one of the largest in the Kakheti Region (eastern Georgia), which currently drains into the Mingachevir Reservoir. The first detailed ichthyological research on this river was conducted in the 1950s and 20 fish species were recorded (Elanidze 1951). The same author noted that there were no native goby species found in the Alazani River and, in his view, the distribution area of Kura goby - *P.cyrius* did not reach as far as Alazani. Since then, sporadic ichthyological investigations have been conducted (Epitashvili et al. 2020) and there are about 30 fish species currently known for the river, including the invasive Amur goby – *Rhinogobiuslindbergi* Berg, 1933.

All along the Alazani River, floodplain riparian forests have developed where small streams originate drained from the surrounding mountains. Such tributaries are often called “Psha” amongst Georgian locals (which means cold clear stream). The water temperatures in these Pshas are constant throughout the year and thus create perfect living conditions for many aquatic species. The biodiversity of these streams is generally poorly understood.

## Materials and methods

**Data collection**

During the regular ichthyological fieldwork in August 2020, one of those Psha, Tsitsmatiant Psha, was sampled and specimens of gobies of the genus *Ponticola* were collected. Fish were caught using the electrofishing device EFGI 650 (http://www.electric-fishing.de/efgi6_e.html) under a permission from the Ministry of Agriculture and Nature Protection of Georgia (permission number: 5768/01). In total, five specimens of gobies were collected in the location and after anaesthesia with MS-222 of a subsample of the collected fishes, a fin-clip was taken and stored in 99.9% molecular grade ethanol for molecular genetic study and specimens were fixed in 70% ethanol for morphological investigation. In 2020-2022, we also conducted three additional field surveys in different seasons at the Tsitsmatiant Psha and collected an additional 15 goby specimens and preserved these in the same way. The collected specimens are kept in the collection of Hydrobiology and Ichthyology Department of the Institute of Zoology of Ilia State University under the instituional code HID-ISU.

**DNA barcoding**

The genetic study was conducted under the ongoing project - Caucasus Barcode of Life (CaBOL - https://ggbc.eu/) with standard CaBOL protocol. In particular, genomic DNA was extracted from pectoral fin clips using a BioSprint96 magnetic bead extractor (Qiagen, Hilden, Germany). PCRs, targeting the standard DNA barcode region COI, were carried out in 20 μl reaction volumes including 2 μl undiluted DNA template, 0.8 μl of each primer (10 pmol/μl; LCO1490-JJ: 5'-CHACWAAYCATAAAGATATYGG-3' and HCO2198-JJ: 5'-AWACTTCVGGRTGVCCAAARAATCA-3', (Astrin and Stüben 2008, Astrin et al. 2016)), 2 μl ‘Q-Solution’ and 10 μl ‘Multiplex PCR Master Mix’ (Qiagen, Hilden, Germany). Cycler temperatures for PCR reaction: thermal conditions included denaturation at 95°C for 1 min, followed by first cycle set (15 cycles): 94°C for 30 sec, annealing at 55°C for 1 min (−1°C per cycle) and extension at 72°C for 1:30 min. Second cycles set (25 cycles): 94°C for 35 sec, 45°C for 1 min, 72°C for 1:30 min, followed by one cycle at 72°C for 3 min and final extension step at 72°C for 5 min. Purification of PCR products and bidirectional sequencing were conducted at either BGI (Hong Kong, China) or Macrogen Europe Laboratories using the amplification primers. Voucher specimens are kept in the ichthyological collections at the Institute of Zoology of Ilia State University (ISU). Extracted genomic DNA is deposited in the ISU Biobank.

**Molecular data analysis**

Data processing and sequence assembly were performed using the Geneious Pro v.7 (Drummond et al. 2011) and the Muscle algorithm (Edgar 2004) was used to align the DNA barcodes after screening for indels or stop codons (Epitashvili et al. 2020). All newly-generated DNA sequences with acceptable quality (with less than 1% ambiguous bases and free of stop codons) were submitted to the Barcode of Life Data System (BOLD, http://v4.boldsystems.org/), including relevant metadata, where they were automatically assigned Barcode Index Numbers (BINs). The sequences will be accessed via the dataset “Freshwater Fishes of Georgia” (SCCBO) in the BOLD Systems.

In addition to the newly-generated DNA barcodes, we included all published DNA barcodes of *Ponticola* spp. deposited at BOLD and NCBI GenBank (Table 1) that originated from Georgia and the Ponto-Caspian biogeographic region. As outgroups, *Neogobiusfluviatilis* (accession number: MW564554) and *Proterorhinus* sp. (accession number: MW564522) were retrieved from GenBank (Epitashvili et al. 2020).

**Figure 1.**
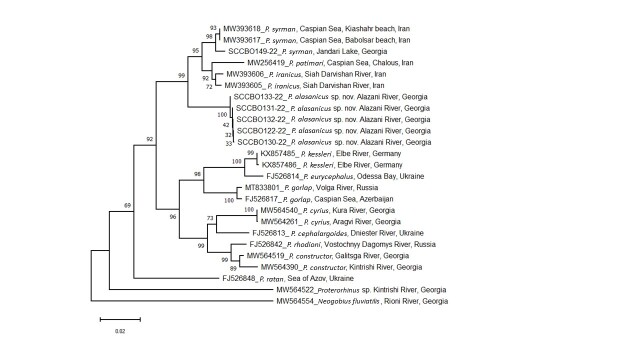
Phylogenetic relationships are presented by the Neighbour-Joining tree, based on the mitochondrial COI barcode region using the Kimura 2 Parameter (K2P) distance model with other default parameters provided by Mega 11 software. Numbers near nodes indicate bootstrap support values from 1000 replicates. The analyses involved 25 COI nucleotide sequences of 14 goby species from the Black, Azov and Caspian Sea Basins.

Subsequently, we evaluated sequence divergence and relationships between *Ponticola* spp, based on uncorrected *p*-distance. A Neighbour-Joining tree based on K2P (Kimura 2-parameter model) distances with 1000 bootstrap replicates was constructed to visualise the phylogenetic relationships amongst specimens (Fig. 1). Analyses were performed using MEGA 11 software (Tamura et al. 2021) and statistical tools provided by BOLD Systems (Ratnasingham and Hebert 2007).

**Morphological examinations**

All measurements were made using a digital caliper with a precision of 0.1 mm. We used a character set commonly used for the study of the Caucasian gobies (Vasil′eva and Vasil′ev 1994a). For counts and meristic and plastic measurements, we also followed methodologies given in Kottelat and Freyhof (2007), Armbruster (2012), Vasil'eva et al. (2015) and Eagderi et al. (2020). The last two branched rays articulating on a single pterygiophore in the second dorsal (D2) and anal (A) fins are noted as “1½”. For the lateral line system, we used terminology from Miller (1986). We calculated mean values and standard deviations for the sample, including the holotype and four paratypes, on all morphometric characters for comparative morphological analysis (see Table 2).

**Comparative materials**: ***Ponticolasyrman***: ISU - SCCBO149-22 – 1007278, 168 mm SL, 205 mm TL, 1, Georgia: Caspian Sea Basin, Jandari Lake, Kvemo Kartli Region, N 41.438641 E 45.208572.

***Ponticolacyrius***: ISU - HIDPcy1, 54-99 mm SL, 65-122 mm TL, 5, Georgia: Caspian Sea Basin, Kvabliani River, Samtskhe-Javakheti Region, N 41.656865 E 42.776685.

ISU - HIDPcy10, 41-105 mm SL, 51-126 mm TL, 5, Georgia: Caspian Sea Basin, Kura River, Samtskhe-Javakheti Region, N 41.322334 E 43.229994.

ISU - HIDPcy13, 74 mm SL, 91 mm TL, 1, Georgia: Caspian Sea Basin, Aragvi River, Mtskheta-Mtianeti Region, N 42.035527 E 44.749659.

***Ponticolaconstructor***: ISU – HIDPco2, 47-77 mm SL, 57.5-92 mm TL, 6, Georgia: Black Sea Basin, Tskaltsitela River, Imereti Region, N 42.269336 E 42.741677.

ISU - HIDPco8, 46-69 mm SL, 56.5-85 mm TL, 3, Georgia: Black Sea Basin, Sulori River, Imereti Region, N 42.081774 E 42.522275.

ISU - HIDPco11, 40-88.5 mm SL, 46-104.5 mm TL, 3, Georgia: Black Sea Basin, Charnali River, Adjara Region, N 41.582625, E 41.625455.

**Abbreviations used**: TL, total length; SL, standard length; HL, lateral head length.

**Collection codes**: ISU – HIDPcy2, Ilia State University – Hydrobiology and Ichthyology Department.

## Data resources

In total, we obtained mitochondrial cytochrome oxidase subunit I (CO1) sequences from five specimens of *Ponticolaalasanicus* sp. n. collected in the Tsitsmatiant Psha stream (right tributary of the Alazani River). For molecular analysis, we downloaded COI sequences of *P.cyrius*, *P.constructor*, *P.syrman*, *P.gorlap*, *P.iranicus*, *P.patimari*, *P.kessleri*, *P.ratan*, *P.rhodioni*, *P.cephalargoides* and *P.eurycephalus* (Table 1). COI sequences of *Neogobiusfluviatilis* and *Proterorhinus* sp. from the Georgian Black Sea Basin (Epitashvili et al. 2020) were used as outgroups (Fig. 1). A Neighbour-Joining phylogenetic tree, based on COI sequences, placed *Ponticolaalasanicus* sp. n. in a group that includes *P.syrman*, *P.iranicus* and *P.patimari*, making it the fourth species, in the so-called *P.syrman* group (for more details see Eagderi et al. (2020)). The new species is separated by a minimum K2P distance of 3.5, 3.6 and 4.8%, from *P.syrman*, *P.iranicus* and *P.patimari*, respectively (Table 3).

In the following, we provide a morphological description of the new *Ponticola* species.

## Taxon treatments

### Ponticola alasanicus sp. n.

DF4A3686-4CA6-542A-BFBF-68909B42B392

5BC69115-FABA-4455-B10F-E3D3E28207AA

https://pubmed.ncbi.nlm.nih.gov/36177146/

#### Materials

- **Type status:** Holotype. **Occurrence:** catalogNumber: HIDPal1; occurrenceRemarks: Captured during the electro fishing; recordedBy: Giorgi Epitashvili; individualID: HIDPal1; individualCount: 1; sex: male; lifeStage: adult; establishmentMeans: native; occurrenceStatus: present; preparations: fin clip; previousIdentifications: *Ponticolacyrius*; associatedSequences: BOLD:AEO718; occurrenceID: 90162319-7839-579C-9EB1-E1F5C8E0D687; **Taxon:** taxonID: SCCBO130-22 – 1007251; scientificName: *Ponticolaalasanicus* Epitashvili, Japoshvili & Mumladze, 2023; originalNameUsage: *Ponticolaalasanicus*; kingdom: Animalia; phylum: Chordata; class: Actinopterygii; order: Gobiiformes; family: Gobiidae; genus: Ponticola; taxonRank: Species; scientificNameAuthorship: Epitashvili, Japoshvili and Mumladze 2023; vernacularName: Alazani goby; **Location:** locationID: Alazani River; higherGeographyID: Kakheti Region; higherGeography: Caucasus; continent: Eurasia; waterBody: Western Caspian Sea Basin; country: Georgia; countryCode: GE; stateProvince: Kakheti; county: Telavi; municipality: Telavi; locality: Shakriani; verbatimLocality: Telavi-Eniseli connecting bridge; maximumElevationInMeters: 347; verbatimLatitude: 41.988808; verbatimLongitude: 45.577726; **Identification:** identifiedBy: Giorgi Epitashvili; dateIdentified: Bella Japoshvili; Levan Mumladze; identificationRemarks: DNA barcoding; **Event:** samplingEffort: 1; eventDate: 11-Aug-2020; year: 2020; month: August; day: 11; habitat: River; **Record Level:** type: PhysicalObject; language: EN; rightsHolder: Ilia State University; institutionID: ISU Ilia State University; collectionCode: Actinopterygii; ownerInstitutionCode: ISU
- **Type status:** Paratype. **Occurrence:** catalogNumber: HIDPal1; occurrenceRemarks: Captured during the electro fishing; recordedBy: Giorgi Epitashvili; individualID: HIDPal1; individualCount: 1; establishmentMeans: native; occurrenceStatus: present; preparations: fin clip; previousIdentifications: *Ponticolacyrius*; associatedSequences: BOLD:AEO718; occurrenceID: 503A9C0F-959F-5928-BBB4-2D352EADA131; **Taxon:** taxonID: SCCBO122-22 – 1007242; scientificName: *Ponticolaalasanicus* Epitashvili, Japoshvili & Mumladze, 2023; originalNameUsage: *Ponticolaalasanicus*; kingdom: Animalia; phylum: Chordata; class: Actinopterygii; order: Gobiiformes; family: Gobiidae; genus: Ponticola; taxonRank: Species; vernacularName: Alazani goby; **Location:** locationID: Alazani River; higherGeographyID: Kakheti Region; higherGeography: Caucasus; continent: Eurasia; waterBody: Western Caspian Sea Basin; country: Georgia; countryCode: GE; stateProvince: Kakheti; municipality: Telavi; locality: Shakriani; verbatimLocality: Telavi-Eniseli connecting bridge; maximumElevationInMeters: 347; verbatimLatitude: 41.988808; verbatimLongitude: 45.577726; **Identification:** identifiedBy: Giorgi Epitashvili; identificationRemarks: DNA barcoding; **Event:** samplingEffort: 1; eventDate: 11-Aug-2020; year: 2020; month: August; day: 11; habitat: River; **Record Level:** type: PhysicalObject; language: EN; rightsHolder: Ilia State University; institutionID: ISU Ilia State University; collectionCode: Actinopterygii; ownerInstitutionCode: ISU
- **Type status:** Paratype. **Occurrence:** catalogNumber: HIDPal1; occurrenceRemarks: Captured during the electro fishing; recordedBy: Giorgi Epitashvili; individualID: HIDPal1; individualCount: 1; establishmentMeans: native; occurrenceStatus: present; preparations: fin clip; previousIdentifications: *Ponticolacyrius*; associatedSequences: BOLD:AEO718; occurrenceID: EB90F6D3-169C-56EB-9E5B-68C90403BEDE; **Taxon:** taxonID: SCCBO131-22 – 1007252; scientificName: *Ponticolaalasanicus* Epitashvili, Japoshvili & Mumladze, 2023; originalNameUsage: *Ponticolaalasanicus*; kingdom: Animalia; phylum: Chordata; class: Actinopterygii; order: Gobiiformes; family: Gobiidae; genus: Ponticola; taxonRank: Species; vernacularName: Alazani goby; **Location:** locationID: Alazani River; higherGeographyID: Kakheti Region; higherGeography: Caucasus; continent: Eurasia; waterBody: Western Caspian Sea Basin; country: Georgia; countryCode: GE; stateProvince: Kakheti; municipality: Telavi; locality: Shakriani; verbatimLocality: Telavi-Eniseli connecting bridge; maximumElevationInMeters: 347; verbatimLatitude: 41.988808; verbatimLongitude: 45.577726; **Identification:** identifiedBy: Giorgi Epitashvili; identificationRemarks: DNA barcoding; **Event:** samplingEffort: 1; eventDate: 11-Aug-2020; year: 2020; month: August; day: 11; habitat: River; **Record Level:** type: PhysicalObject; language: EN; rightsHolder: Ilia State University; institutionID: ISU Ilia State University; collectionCode: Actinopterygii; ownerInstitutionCode: ISU
- **Type status:** Paratype. **Occurrence:** catalogNumber: HIDPal1; occurrenceRemarks: Captured during the electro fishing; recordedBy: Giorgi Epitashvili; individualID: HIDPal1; individualCount: 1; establishmentMeans: native; occurrenceStatus: present; preparations: fin clip; previousIdentifications: *Ponticolacyrius*; associatedSequences: BOLD:AEO718; occurrenceID: D59DD48D-957E-5FA7-BCB6-CAD685B8C637; **Taxon:** taxonID: SCCBO132- 22 – 1007253; scientificName: *Ponticolaalasanicus* Epitashvili, Japoshvili & Mumladze, 2023; originalNameUsage: *Ponticolaalasanicus*; kingdom: Animalia; phylum: Chordata; class: Actinopterygii; order: Gobiiformes; family: Gobiidae; genus: Ponticola; taxonRank: Species; vernacularName: Alazani goby; **Location:** locationID: Alazani River; higherGeographyID: Kakheti Region; higherGeography: Caucasus; continent: Eurasia; waterBody: Western Caspian Sea Basin; country: Georgia; countryCode: GE; stateProvince: Kakheti; municipality: Telavi; locality: Shakriani; verbatimLocality: Telavi-Eniseli connecting bridge; maximumElevationInMeters: 347; verbatimLatitude: 41.988808; verbatimLongitude: 45.577726; **Identification:** identifiedBy: Giorgi Epitashvili; identificationRemarks: DNA barcoding; **Event:** samplingEffort: 1; eventDate: 11-Aug-2020; year: 2020; month: August; day: 11; habitat: River; **Record Level:** type: PhysicalObject; language: EN; rightsHolder: Ilia State University; institutionID: ISU Ilia State University; collectionCode: Actinopterygii; ownerInstitutionCode: ISU
- **Type status:** Paratype. **Occurrence:** catalogNumber: HIDPal1; occurrenceRemarks: Captured during the electro fishing; recordedBy: Giorgi Epitashvili; individualID: HIDPal1; individualCount: 1; establishmentMeans: native; occurrenceStatus: present; preparations: fin clip; previousIdentifications: *Ponticolacyrius*; associatedSequences: BOLD:AEO718; occurrenceID: 8F567408-B657-5672-9F93-920C883F5ACC; **Taxon:** taxonID: SCCBO133-22 – 1007254; scientificName: *Ponticolaalasanicus* Epitashvili, Japoshvili & Mumladze, 2023; originalNameUsage: *Ponticolaalasanicus*; kingdom: Animalia; phylum: Chordata; class: Actinopterygii; order: Gobiiformes; family: Gobiidae; genus: Ponticola; taxonRank: Species; vernacularName: Alazani goby; **Location:** locationID: Alazani River; higherGeographyID: Kakheti Region; higherGeography: Caucasus; continent: Eurasia; waterBody: Western Caspian Sea Basin; country: Georgia; countryCode: GE; stateProvince: Kakheti; municipality: Telavi; locality: Shakriani; verbatimLocality: Telavi-Eniseli connecting bridge; maximumElevationInMeters: 347; verbatimLatitude: 41.988808; verbatimLongitude: 45.577726; **Identification:** identifiedBy: Giorgi Epitashvili; identificationRemarks: DNA barcoding; **Event:** samplingEffort: 1; eventDate: 11-Aug-2020; year: 2020; month: August; day: 11; habitat: River; **Record Level:** type: PhysicalObject; language: EN; rightsHolder: Ilia State University; institutionID: ISU Ilia State University; collectionCode: Actinopterygii; ownerInstitutionCode: ISU
- **Type status:** Other material. **Occurrence:** catalogNumber: HIDPcy2; occurrenceRemarks: Captured during the electro fishing; recordedBy: Giorgi Epitashvili; individualID: HIDPcy2; individualCount: 1; sex: male; lifeStage: adult; establishmentMeans: native; occurrenceStatus: present; preparations: fin clip; previousIdentifications: *Ponticolacyrius*; associatedSequences: BOLD:AEO718; occurrenceID: A8526592-C88A-5387-B64A-B3464AC36F6A; **Taxon:** taxonID: HIDPcy2; scientificName: *Ponticolaalasanicus* Epitashvili, Japoshvili & Mumladze, 2023; originalNameUsage: *Ponticolaalasanicus*; kingdom: Animalia; phylum: Chordata; class: Actinopterygii; order: Gobiiformes; family: Gobiidae; genus: Ponticola; taxonRank: Species; vernacularName: Alazani goby; **Location:** locationID: Alazani River; higherGeographyID: Kakheti Region; higherGeography: Caucasus; continent: Eurasia; waterBody: Western Caspian Sea basin; country: Georgia; countryCode: GE; stateProvince: Kakheti; municipality: Telavi; locality: Shakriani; verbatimLocality: Telavi-Eniseli connecting bridge; maximumElevationInMeters: 347; verbatimLatitude: 41.988808; verbatimLongitude: 45.577726; **Identification:** identifiedBy: Giorgi Epitashvili; identificationRemarks: Morphology & DNA barcoding; **Event:** samplingEffort: 1; eventDate: 26-Dec-2020; year: 2020; month: December; day: 26; habitat: River; **Record Level:** type: PhysicalObject; language: EN; rightsHolder: Ilia State University; institutionID: ISU Ilia State University; collectionCode: Actinopterygii; ownerInstitutionCode: ISU
- **Type status:** Other material. **Occurrence:** catalogNumber: HIDPal2; occurrenceRemarks: Captured during the electro fishing; recordedBy: Giorgi Epitashvili; individualID: HIDPal2; individualCount: 1; sex: female; lifeStage: adult; establishmentMeans: native; occurrenceStatus: present; preparations: fin clip; previousIdentifications: *Ponticolacyrius*; associatedSequences: BOLD:AEO718; occurrenceID: 81E8A0FB-89DE-519E-90A2-32214DE555F6; **Taxon:** taxonID: HIDPal2; scientificName: *Ponticolaalasanicus* Epitashvili, Japoshvili & Mumladze, 2023; originalNameUsage: *Ponticolaalasanicus*; kingdom: Animalia; phylum: Chordata; class: Actinopterygii; order: Gobiiformes; family: Gobiidae; genus: Ponticola; taxonRank: Species; vernacularName: Alazani goby; **Location:** locationID: Alazani River; higherGeographyID: Kakheti Region; higherGeography: Caucasus; continent: Eurasia; waterBody: Western Caspian Sea Basin; country: Georgia; countryCode: GE; stateProvince: Kakheti; municipality: Telavi; locality: Shakriani; verbatimLocality: Telavi-Eniseli connecting bridge; maximumElevationInMeters: 347; verbatimLatitude: 41.988808; verbatimLongitude: 45.577726; **Identification:** identifiedBy: Giorgi Epitashvili; identificationRemarks: Morphology & DNA barcoding; **Event:** samplingEffort: 1; eventDate: 26-Dec-2020; year: 2020; month: December; day: 26; habitat: River; **Record Level:** type: PhysicalObject; language: EN; rightsHolder: Ilia State University; institutionID: ISU Ilia State University; collectionCode: Actinopterygii; ownerInstitutionCode: ISU
- **Type status:** Other material. **Occurrence:** catalogNumber: HIDPal2; occurrenceRemarks: Captured during the electro fishing; recordedBy: Giorgi Epitashvili; individualID: HIDPal2; individualCount: 1; sex: female; lifeStage: adult; establishmentMeans: native; occurrenceStatus: present; preparations: fin clip; previousIdentifications: *Ponticolacyrius*; associatedSequences: BOLD:AEO718; occurrenceID: 0BE084FB-16C0-5895-B408-4962BAF78837; **Taxon:** taxonID: HIDPal2; scientificName: *Ponticolaalasanicus* Epitashvili, Japoshvili & Mumladze, 2023; originalNameUsage: *Ponticolaalasanicus*; kingdom: Animalia; phylum: Chordata; class: Actinopterygii; order: Gobiiformes; family: Gobiidae; genus: Ponticola; taxonRank: Species; vernacularName: Alazani goby; **Location:** locationID: Alazani River; higherGeographyID: Kakheti Region; higherGeography: Caucasus; continent: Eurasia; waterBody: Western Caspian Sea basin; country: Georgia; countryCode: GE; stateProvince: Kakheti; municipality: Telavi; locality: Shakriani; verbatimLocality: Telavi-Eniseli connecting bridge; maximumElevationInMeters: 347; verbatimLatitude: 41.988808; verbatimLongitude: 45.577726; **Identification:** identifiedBy: Giorgi Epitashvili; identificationRemarks: Morphology & DNA barcoding; **Event:** samplingEffort: 1; eventDate: 30-May-2022; year: 2022; month: May; day: 30; habitat: River; **Record Level:** type: PhysicalObject; language: EN; rightsHolder: Ilia State University; institutionID: ISU Ilia State University; collectionCode: Actinopterygii; ownerInstitutionCode: ISU
- **Type status:** Other material. **Occurrence:** catalogNumber: HIDPal2; occurrenceRemarks: Captured during the electro fishing; recordedBy: Giorgi Epitashvili; individualID: HIDPal2; individualCount: 1; sex: female; lifeStage: adult; reproductiveCondition: pregnant; establishmentMeans: native; occurrenceStatus: present; preparations: fin clip; previousIdentifications: *Ponticolacyrius*; associatedSequences: BOLD:AEO718; occurrenceID: 60686D75-310B-5299-86C3-4F1010A91CFA; **Taxon:** taxonID: HIDPal2; scientificName: *Ponticolaalasanicus* Epitashvili, Japoshvili & Mumladze, 2023; originalNameUsage: *Ponticolaalasanicus*; kingdom: Animalia; phylum: Chordata; class: Actinopterygii; order: Gobiiformes; family: Gobiidae; genus: Ponticola; taxonRank: Species; vernacularName: Alazani goby; **Location:** locationID: Alazani River; higherGeographyID: Kakheti Region; higherGeography: Caucasus; continent: Eurasia; waterBody: Western Caspian Sea Basin; country: Georgia; countryCode: GE; stateProvince: Kakheti; municipality: Telavi; locality: Shakriani; verbatimLocality: Telavi-Eniseli connecting bridge; maximumElevationInMeters: 347; verbatimLatitude: 41.988808; verbatimLongitude: 45.577726; **Identification:** identifiedBy: Giorgi Epitashvili; identificationRemarks: Morphology & DNA barcoding; **Event:** samplingEffort: 1; eventDate: 30-May-2022; year: 2022; month: May; day: 30; habitat: River; **Record Level:** type: PhysicalObject; language: EN; rightsHolder: Ilia State University; institutionID: ISU Ilia State University; collectionCode: Actinopterygii; ownerInstitutionCode: ISU
- **Type status:** Other material. **Occurrence:** catalogNumber: HIDPal2; occurrenceRemarks: Captured during the electro fishing; recordedBy: Giorgi Epitashvili; individualID: HIDPal2; individualCount: 1; sex: male; lifeStage: adult; establishmentMeans: native; occurrenceStatus: present; preparations: whole specimen; previousIdentifications: *Ponticolacyrius*; associatedSequences: BOLD:AEO718; occurrenceID: 2C25F16A-0C05-55B3-9192-CD0F8FD82059; **Taxon:** taxonID: HIDPal2; scientificName: *Ponticolaalasanicus* Epitashvili, Japoshvili & Mumladze, 2023; originalNameUsage: *Ponticolaalasanicus*; kingdom: Animalia; phylum: Chordata; class: Actinopterygii; order: Gobiiformes; family: Gobiidae; genus: Ponticola; taxonRank: Species; vernacularName: Alazani goby; **Location:** locationID: Alazani River; higherGeographyID: Kakheti Region; higherGeography: Caucasus; continent: Eurasia; waterBody: Western Caspian Sea Basin; country: Georgia; countryCode: GE; stateProvince: Kakheti; municipality: Telavi; locality: Shakriani; verbatimLocality: Telavi-Eniseli connecting bridge; maximumElevationInMeters: 347; verbatimLatitude: 41.988808; verbatimLongitude: 45.577726; **Identification:** identifiedBy: Giorgi Epitashvili; identificationRemarks: Morphology & DNA barcoding; **Event:** samplingEffort: 1; eventDate: 30-May-2022; year: 2022; month: May; day: 30; habitat: River; **Record Level:** type: PhysicalObject; language: EN; rightsHolder: Ilia State University; institutionID: ISU Ilia State University; collectionCode: Actinopterygii; ownerInstitutionCode: ISU

#### Description

**Holotype**: ISU - SCCBO130-22 – 1007251; Male, 73.5 mm TL, 61.0 mm SL; Georgia, Kakheti Region, Western Caspian Sea Basin, Tsitsmatiant Psha, right tributary of the Alazani River, near the Telavi-Eniseli connecting bridge, N41.988808, E45.577726; (collecting date: 11-Aug-2020).

**Paratypes**: ISU - SCCBO122-22 – 1007242, ISU - SCCBO131-22 – 1007252, ISU - SCCBO132- 22 – 1007253, ISU - SCCBO133-22 – 1007254, 52.0 – 71.0 mm TL, 42.0 – 59.0 mm SL, same data as holotype.

**Additional materials used in morphological analyses**: ISU - HIDPcy2, male, 71.0 mm TL, 58.5 mm SL; ISU – HIDPal2, female, 45.0 mm TL, 38.0 mm SL; ISU – HIDPal2, female, 69.0 mm TL, 57.0 mm SL; ISU – HIDPal2, female, 63.0 mm TL, 52 mm SL; HID – Pal2, male, 74.0 mm TL, 59.0 mm SL, same data as holotype/paratypes. **Remark**: these specimens were captured during resampling in 26-Dec-2020 and 30-May-2022 in the same location.

**Description**: D_1_ VI, D_2_ I / 16½, A I / 12½, l.l. 53. Body laterally compressed, gradually tapering from head to tail; minimum body depth almost 1.4 times less than caudal peduncle length; lateral parts of the body covered by ctenoid scales; first and second dorsal fins almost touching with their bases; second dorsal fin of uniform height; head large, depressed, wider than deep, its length approaches almost 3.4th its SL; nape scaled completely; cycloid scales cover upper part of opercle, cheeks noticeably swollen; snout longer than eye; eye diameter 4.5 times its HL; lower jaw slightly protruding; upper lip is uniform; pelvic disc elongated, not concave but flat, its length about 1.6 times less than ventro-anal distance, not reaching anus; pectoral fins reach base of first dorsal branched ray; caudal fin rounded.

**Figure 2.**
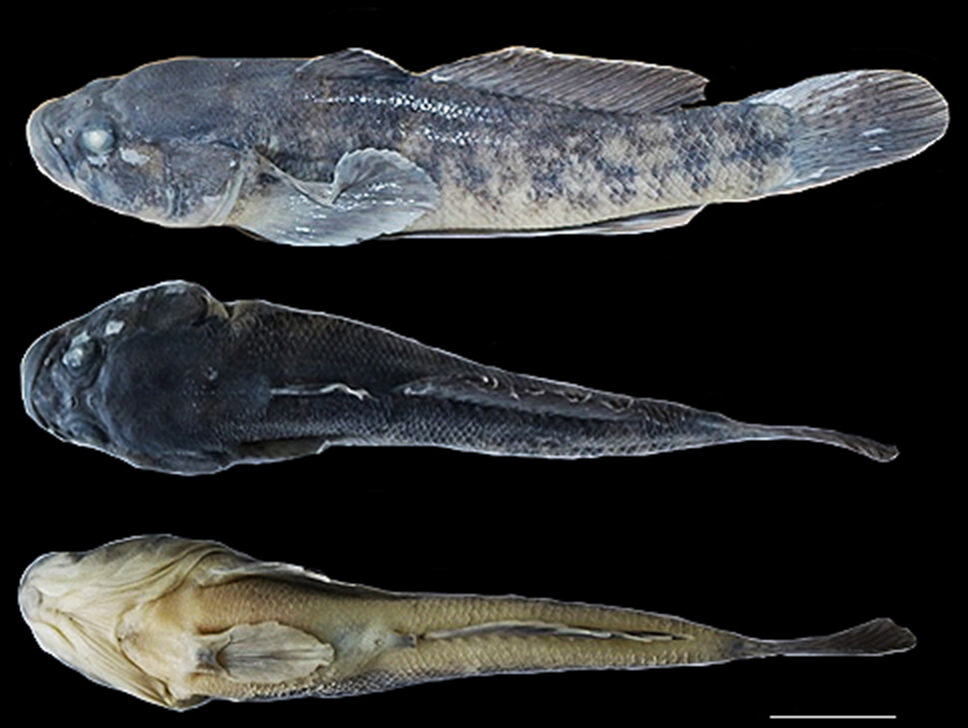
*Ponticolaalasanicus* sp. n. holotype; ISU - SCCBO130-22 – 1007251, male, 73.5 mm TL, Georgia, Tsitsmatiant Psha (Alazani River Basin). Scale = 1 cm.

The colouration of the preserved holotype yellowish-grey with dark brown and black blotches on the body (Fig. 2). The upper part of the head is dark, the throat white with yellow-marble colouration; the same colouration is present in the upper and lower parts of the whole body; on the last part of the caudal peduncle, there is a dark spot in the shape of a cross; the predorsal area is linearly concave in the middle with dark brown and black scales; vertically arranged uneven spots follow the lateral sides of the whole body; ventral disc with white/marble colouration, pectoral, dorsal, anal and caudal fins yellowish-grey; first and second dorsal-fin with some brown stripes and blotches; first three unbranched dorsal fin rays with dark stripes and, in upper part, transparent/white; dark brown spots on the upper part of the pectoral fin base. Body proportions and morphometric characters are given in Table 2.

**Extended description based on all type specimens**: Overall meristic characteristics: D_1_ VI-VII, D_2_ I / 15½-16½, A I / 10 ½-12½; l.l. 48-55. All studied specimens have a relatively short body, laterally compressed at caudal peduncle, the maximum length (TL) was fixed as 73.5 mm in holotype; 15½-16½ branched dorsal rays; 10½-12½ branched anal rays; scales in lateral line series from 48 to 55; minimum body depth is 1.3–1.5 times its caudal peduncle length; head relatively large, depressed, wider than deep, its length 3.1-3.7 times its SL; head width slightly larger than depth at nape, interorbital distance short from 2.0 to 3.0 mm; lower jaw slightly protruded, upper and low jaws same length; lips almost uniform, upper lip slightly swollen; pelvic disc short, elongated and flat, not reaching anus. In our samples, the largest specimen was a male with 59.0 mm SL and 74.0 mm TL. Sexual dimorphism is not pronounced, except that males are slightly longer than females.

**Figure 3.**
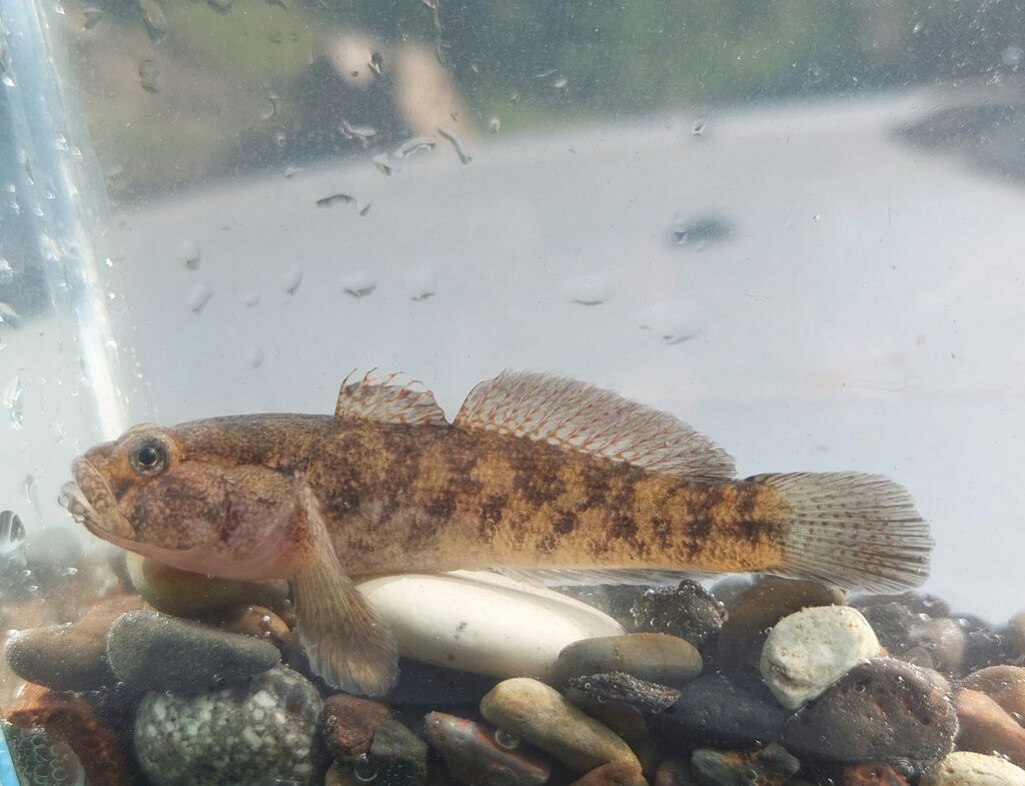
Live specimen of *Ponticolaalasanicus* sp. n. from the type location.

**Colouration**: Live specimens have yellowish-grey colour with dark brown and black blotches on the body (Fig. 3). Some goby species, for example, *P.hircaniaensis* (Zarei et al. 2022) distributed in the southern Caspian Basin have a similar colouration. Studied specimens after fixation in 96% molecular grade ethanol are usually yellowish-grey with dark brown and black blotches on the body. Some specimens also have brownish colouration on the head. The abdomen is white/marbled. Pelvic disc with white/grey colouration; pectoral, dorsal, anal and caudal fins yellowish-grey; first and second dorsal-fin with some brown/dark or grey stripes and blotches; first dorsal fin ray with dark stripes and, in upper part, transparent/white in some specimens; dark brown spots on the upper part of the pectoral fin base.

**Figure 4.**
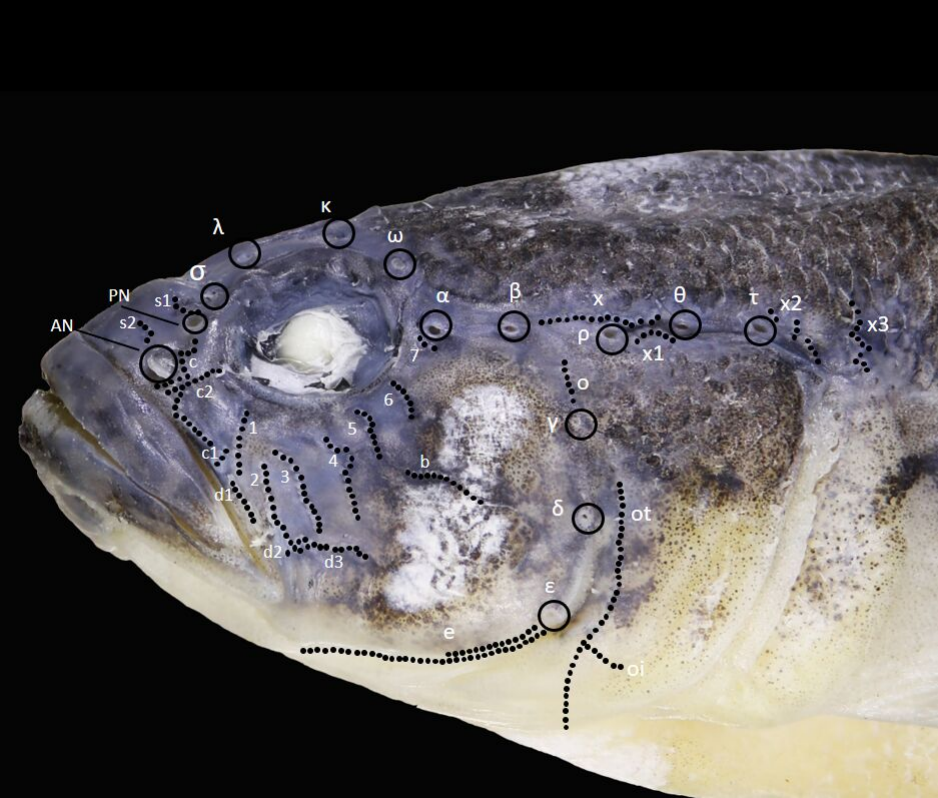
Diagram of head lateral-line sensory system in the holotype of *Ponticolaalasanicus* sp. n.; pores are designated by Greek letters, rows of neuromasts (genipores/sensory papillae) by Latin letters and Arabic numerals; AN - anterior nostril, PN - posterior nostril.

**Head lateral-line system** in *Ponticolaalasanicus* sp. n. is similar to the other known species from the genus *Ponticola* (Fig. 4): neuromast organs form seven transverse rows, four (*1*-*4*) before, one (*5*) in parallel and two (*6* and *7*) above hyomandibular row *b*, row 7 consists of several papillae before anterior oculoscapular pore α; three (C, C_1_ and C_2_) under PN and lacking row *a*. One hyomandibular row (S_1_) above PN and another one (S_2_) above AN, respectively. Lacking papillae row *5i* and *6i*. Row *5* and *6* long, papillae row (*O*) above oculoscapular pore γ. Sub-orbital longitudinal row *d* consists of three parts: the anterior *d_1_* oblique, following the border of the upper lip and not reaching below the anterior origin of *d_2_*, the posterior longitudinal row *d_2_* and *d_3_* are related to each other. Anterior and posterior oculoscapular canals and pre-opercular canals present with pores σ, λ, κ ω, α, β, ρ, θ, τ and γ, δ, ε, respectively. Anterior oculoscapular pore ρ and posterior oculoscapular pore θ well-separated, row X_1_ between them, row X starts from pore β and reaches pore θ.

#### Diagnosis

D_1_ – VI, D_2_ I / 15½-16½, A I / 10½ – 12½; nape scaled completely, scales cycloid and ctenoid, cycloid scales covering upper part of opercle; lateral line system with sub-orbital row *d* continuous; predorsal area is linearly concave in middle; first dorsal fin with oblique black stripe between first two or three rays, the tip of the first 3-4 rays is transparent white; species has one large dark spot at the base of the pectoral fin; ventral disc has oval/elongated shape, short, not reaching anus.

##### Differential diagnosis

*Ponticolaalasanicus* sp. n. is distinguished from other species of the genus *Ponticola* entering freshwater habitats in the Caspian Sea Basin by the following characters:

It can be distinguished from *P.syrman* by fewer scales in mid-lateral series (48-55 vs. 57-67+2-3); relatively short body (maximum size 73.5 mm TL vs. 220 mm TL); cycloid scales on the nape (vs. ctenoid scales); the ventral disc is slightly shorter (16.7 -19.7 vs. 19.0-20.0% SL) and shorter pre-anal distance (56.7-60.8 vs. 61.3-64.4% SL); *P.alasanicus* sp. n. does not have transverse infraorbital papillae rows below longitudinal hyomandibular row *b* (vs. *P.syrman* has three rows).

*Ponticolaalasanicus* sp. n. is distinguished from *P.iranicus* by relatively small body-size (42.0-61.0 mm SL vs. 65.0-83.0 mm SL); fewer scales in lateral series (48-55 vs. 55-60); pectoral fin reaching to second dorsal fin (vs. not reaching); minimum body depth almost 1.4 times less than caudal peduncle length (vs. 1.5-1.9 times in *P.iranicus*); interorbital distance particularly large from 1.3 to 1.5 mm in eye diameter compared to 0.3-0.8 mm in *P.iranicus*; absence of transverse infraorbital papillae rows bellow longitudinal hyomandibular row *b* (vs. *P.iranicus* has two rows: *5i* and *6i*).

*Ponticolaalasanicus* sp. n. differs from *P.patimari* by a ventral disc that is noticeably shorter (16.7-19.7 vs. 20.2-26.0% SL), not reaching anus (vs. reaching in some specimens of *P.patimari*); additionally, there are seven transverse rows in *P.alasanicus* sp. n. comapred to six transverse rows in *P.patimari*).

*Ponticolaalasanicus* sp. n. is also distinguished from the recently described *P.hircaniaensis* Zarei, Esmaeili, Kovačić, Schliewen & Abbasi, 2022 from the southern Caspian Sea Basin by a lower number of scales in the mid-lateral series (48-55 vs. 52-59) and a relatively short head depth at nape (43.3-53.1 vs. 70.9-81.0%).

*Ponticolaalasanicus* sp. n. is distinguished from *P.gorlap* by having a longer maximum body depth (21.6-24.6 vs. 15.0-18.1% SL); a longer least depth of caudal peduncle (12.0-14.3 vs. 7.8-9.4% SL); cycloid scales on the nape and predorsal area (vs. ctenoid scales in *P.gorlap*); less scales in mid-lateral series (48-55 vs. 68-72 + 3-4); interorbital distance large (1.3-1.5 times vs. 0.8-0.9 times in eye diameter) and noticeably small body length (vs. TL in *P.gorlap* is almost three times larger).

*Ponticolaalasanicus* sp. n. differs from *P.cyrius* by having an elongated and flattened ventral disc (vs. round and concave ventral disc); anterior membrane of ventral disc with shallow, slightly excised lobes (vs. deeply cut and angular in *P.cyrius*); *P.alasanicus* sp. n. has longer least depth of caudal peduncle (12.0-14.3 vs. 9.4-10.0% SL); shorter head width (66.7-81.2 vs. 81.4-100.0%); predorsal area with dark brown and black colouration (vs. marbled).

*Ponticolaalasanicus* sp. n. is distinguished from all other *Ponticola* species (e.g. *P.constructor*, *P.rhodioni*, *P.rizensis* and *P.turani*) occurring in the Caucasian Black Sea Basin by following characters: *P.alasanicus* has fewer branched rays in dorsal fin 15½-16½ vs. 16½-19½ in *P.constructor* and *P.rhodioni* and 17½-19½ in *P.turani* and *P.rizensis*, respectively. *P.alasanicus* is also easily distinguished from the aforementioned gobies by noticeably short body (maximum reported SL = 61 mm) and elongated, short and flat-shaped ventral disc (vs. round and concave, with relatively long size ventral disc).

#### Etymology

The species is named after the Alazani River.

#### Distribution

*Ponticolaalasanicus* sp. n. is endemic to the Alazani River Basin and currently known from the type locality (Tsitsmatiant Psha) and also from the small swamps near Shakriani (N42.001151 E45.600603) and Mshvidobiani (N41.76147 E46.130432) villages. Most likely inhabiting similar tributaries and habitats of the aforementioned river basin.

#### Ecology

**Figure 5.**
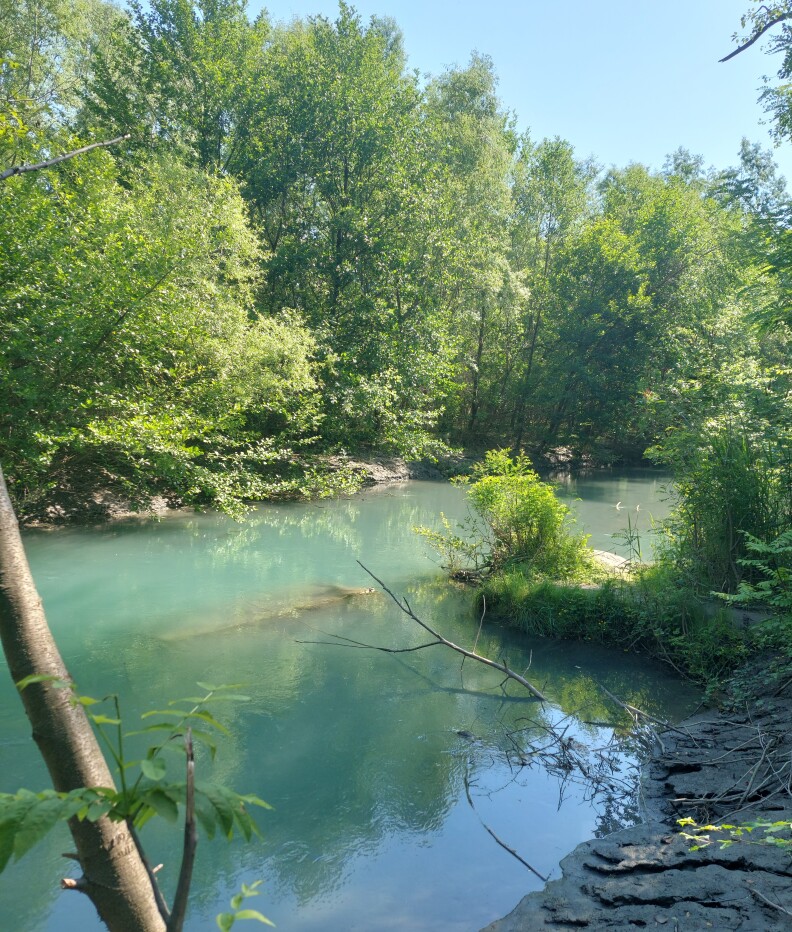
Type location - Tsitsmatiant Psha stream, right tributary of the Alazani River, typical habitat of *Ponticolaalasanicus* sp. n.

The new species inhabits small to medium-sized rivers called “Psha” (which means clear, cold water in Georgian and which can be used for drinking) with sand and plant substrate (Fig. 5). These rivers rise from the ground and are typical for the Mtkvari, Alazani and Iori River lowlands.

#### Conservation

The new species has a small range (i.e. less than 20 km^2^) and is known only from the restricted area of the Alazani River Basin. The surrounding area of the known occurrences is within agricultural land where extensive use of pesticides and fertilisers is common. In addition, water intake from the river and illegal fishing are frequent (personal observation). Thus, we assume strong anthropogenic impacts on the habitat. Currently, the population trend is unknown. However, due to its extremly small range and high anthropogenic pressure, the conservation status of *Ponticolaalasanicus* sp. n. can be regarded as vulnerable (IUCN 2012).

#### Biology

Inhabits lowland rivers and swampy habitats with sand and plant bottom. Often found near submerged trees and roots. Feeds on aquatic invertebrates. Spawns in May-June.

## Discussion

Research on the diversity of freshwater fishes of the South Caucasus Region and particularly in Georgia has been done with more or less intensity in past years. Recent molecular-genetic studies (Epitashvili et al. 2020) and annotated checklist of the freshwater fishes of the South Caucasus Region (Kuljanishvili et al. 2020) have revealed several new species as well as taxonomic rearrangement in freshwater fish fauna of the region. For instance, less than half of the fish species distributed in Georgian inland waters were studied with the aid of molecular genetics methods. Accordingly, it is expected that the fish diversity in Georgia is much higher and this study is one example of that. Therefore, the description of new species is to be expected in the future. Freshwater ecosystems and their organisms are amongst the most vulnerable in the world as well as in Georgia and further studies, including the use of genetic methods, will be important in order to fully understand the diversity of Georgian fishes which will also help us in their protection.

## Supplementary Material

XML Treatment for Ponticola alasanicus

## Figures and Tables

**Table 1.** List of public CO1 sequences used for molecular analyses taken from BOLD and NCBI GenBank with information on Accession Number, drainage and country of origin.

**Species**	**Drainage**	**Published by**	**GenBank/BOLD Acc. N.**
*Ponticolakessleri*	Elbe River (Germany)	Thiel et al. (2017)	KX857485
*P.kessleri*	Elbe River (Germany)	Thiel et al. (2017)	KX857486
*P.ratan*	Sea of Azov (Ukraine)	Neilson and Stepien (2009)	FJ526848
*P.cephalargoides*	Dniester River (Ukraine)	Neilson and Stepien (2009)	FJ526813
*P.rhodioni*	Vostochnyy Dagomys River (Russia)	Neilson and Stepien (2009)	FJ526842
*P.eurycephalus*	Odessa Bay (Ukraine)	Neilson and Stepien (2009)	FJ526814
*P.syrman*	Caspian Sea, Kiashahr beach (Iran)	Zarei et al. (2021)	MW393618
*P.syrman*	Caspian Sea, Babolsar beach (Iran)	Zarei et al. (2021)	MW393617
*P.gorlap*	Volga River (Russia)	Karabanov et al. (2022)	MT833801
*P.gorlap*	Caspian Sea (Azerbaijan)	Neilson and Stepien (2009)	FJ526817
*P.constructor*	Galitsga River, Black Sea basin (Georgia)	Epitashvili et al. (2020)	MW564519
*P.constructor*	Kintrishi River, Black Sea basin (Georgia)	Epitashvili et al. (2020)	MW564390
*P.cyrius*	Kura River (Georgia)	Epitashvili et al. (2020)	MW564540
*P.cyrius*	Aragvi River (Georgia)	Epitashvili et al. (2020)	MW564261
*P.iranicus*	Caspian Sea, Siah Darvishan River (Iran)	Zarei et al. (2021)	MW393605
*P.iranicus*	Caspian Sea, Siah Darvishan River (Iran)	Zarei et al. (2021)	MW393606
*P.patimari*	Caspian Sea, Chalous (Iran)	Eagderi et al. (2020)	MW256419
*Neogobiusfluviatilis*	Rioni River, Black Sea basin (Georgia)	Epitashvili et al. (2020)	MW564554
*Proterorhinus* sp.	Kintrishi River, Black Sea basin (Georgia)	Epitashvili et al. (2020)	MW564522

**Table 2.** Morphometric characters (ranges, means and standard deviations) of type specimens of *Ponticolaalasanicus* sp. n.

	**Holotype + Paratypes (n = 5)**		
	Holotype	Paratypes	
**Character in mm**		Range	Mean±SD
Total body length	73.5	52.0-73.5	65.7±7.86
Standard body length	61.0	42.0-61.0	54.6±7.23
Number of anal fin branched rays	12.0	10.5-12.5	11.7±0.81
Number of the second dorsal fin branched rays	16.0	15.0-16.0	15.5±0.32
**In percent of SL**			
Maximum body depth	23.8	21.6-24.6	23.1± 1.22
Least depth of caudal peduncle	12.9	12.0-14.3	13.04±0.76
Predorsal distance	36.2	35.6-39.3	36.82±1.36
Length of the second dorsal fin base	32.9	29.4-33.3	32.38±1.50
Height of the second dorsal fin	14.4	11.8-14.4	13.04±1.02
Pre-anal distance	57.4	56.7-60.8	58.44±1.71
Length of anal fin base	24.6	21.6-24.6	22.82±1.19
Length of pectoral fin	27.5	22.5-27.5	24.78±1.86
Length of ventral disc	19.7	16.7-19.7	17.96±1.09
Caudal peduncle length	18.0	17.1-19.0	18±0.64
Width of caudal peduncle at the anal fin	6.6	6.6-8.5	7.6±0.67
Minimum width of caudal peduncle	4.1	3.9-4.9	4.38±0.40
Head length	29.5	27.1-32.1	29.28±1.66
**In percent of HL**			
Horizontal diameter of eye	22.2	22.2-25.6	23.24±1.25
Pre-orbital distance	28.3	28.3-31.2	29.7±0.94
Postorbital distance	60.6	55.6-60.6	58.3±2.12
Interorbital distance	16.7	14.8-17.5	16.56±0.93
Width of upper lip	45.0	44.1-48.1	45.66±1.52
Head depth at nape	50.0	43.3-53.1	47.92±3.62
Head width	75.0	66.7-81.2	74.54±4.72

**Table 3.** Estimates of evolutionary divergence (%) over sequence pairs between *Ponticola* species distributed in the south-western Caspian and Georgian Black Sea Basins. Analyses were conducted using the Kimura 2-parameter model.

**Species**	**N**	1	2	3	4	5	6	7
*P.syrman*	**1**							
*P.alasanicus* sp. n.	**2**	3.52						
*P.constructor*	**3**	7.22	8.25					
*P.cyrius*	**4**	7.86	8.47	4.96				
*P.gorlap*	**5**	7.01	8.03	6.78	6.58			
*P.iranicus*	**6**	1.89	3.59	7.74	8.17	7.63		
*P.patimari*	**7**	2.94	4.77	7.84	8.27	7.41	1.95	
